# Integration of Morphology, Histology, and Ultrastructure of the Gall-Inducing *Andricus quercustozae* (Cynipidae) Larval Alimentary Canal

**DOI:** 10.3390/insects17070752

**Published:** 2026-07-22

**Authors:** Sanja Puljas, Ivana Bočina, Nives Kević, Ivica Šamanić, Juraj Kamenjarin, Petar Ćurlin

**Affiliations:** Department of Biology, Faculty of Science, University of Split, Ruđera Boškovića 33, 21000 Split, Croatia; bocina@pmfst.hr (I.B.); nkevic@pmfst.hr (N.K.); isamanic@pmfst.hr (I.Š.); jk@pmfst.hr (J.K.); pcurlin@pmfst.hr (P.Ć.)

**Keywords:** Cynipidae, *Andricus quercustozae*, alimentary canal, biosynthesis, digestive cells

## Abstract

The transition from simple herbivory to specialised gall induction in Cynipidae marks an evolutionary peak in insect–plant interactions. This structural and ultrastructural study suggests that the gall wasp larva has evolved from a passive consumer into a biological architect, utilising a highly specialised body plan. The structure of the large midgut, the midgut epithelium reinforced by septate junctions, and the cuboidal cells of the hindgut allow for the possibility that the larva of *Andricus quercustozae* may control itself independently from its host, working as an active producer rather than just recycling materials. The foregut constitutes approximately 10% of the alimentary canal length, while the large blind midgut occupies 80%. The remaining 10% consists of a specialised hindgut tube, which is anteriorly closed and contains a reinforced, cuticularised external anal pore. This posterior structure, together with a dense aggregation of urocytes and oenocytes in the anal region, presumed systemic homeostasis and the symmetrical distribution of hormonal signals. These anatomical hallmarks provide a structural baseline to hypothesize that biosynthesis may represent a key mechanism for phytohormone acquisition, while suggesting that this closed-circuit anatomy could potentially be a contributing factor influencing the spherical morphogenesis of the gall.

## Simple Summary

This study presents a detailed description of the internal anatomy of the gall-inducing wasp larvae of *Andricus quercustozae*. Key features include a closed midgut occupying most of the body cavity, specialized cell clusters within the fat body, and a well-developed hindgut tube. These anatomical adaptations indicate specialized mechanisms for nutrient storage and regulation of internal pressure, thereby establishing a structural basis for subsequent physiological investigations.

## 1. Introduction

The morphology, histology, and physiology of the insect alimentary canal are closely linked to feeding habits and digestive and absorptive processes [1,2,3,4]. The canal typically has a single layer of epithelial cells, supported by a basal lamina and surrounded by muscle tissue. It consists of two ectodermal regions, the foregut (stomodeum) and hindgut (proctodeum), with the endodermal midgut (mesenteron) between them. In Hymenopterans, gut morphology changes significantly during development to meet the nutritional needs of each life stage [5,6]. Specifically, the larval foregut is simplified, serving mainly as a conduit for nutrient transport and is characterised by a short, tubular structure [7]. As the organism matures, the adult foregut elongates and develops structures such as the crop, which stores and processes liquid foods like nectar [8,9,10]. Within the foregut, the pharynx, oesophagus, and proventriculus each have specific roles: the pharynx, equipped with sensory neurons, assesses food quality; the oesophagus transports food; and the proventriculus regulates entry into the midgut and sorts food by particle size using hair-like projections [11]. Moving to the midgut, this region is the primary site of enzymatic digestion and nutrient absorption, efficiently transferring nutrients to the hemolymph [1,2,4,12]. Significantly, the midgut contains a peritrophic matrix—a robust layer of proteins and chitin fibres, which is absent in the foregut and hindgut. This matrix protects the midgut epithelium from damage by digestive enzymes, physical injury, and harmful microbes [1,2] and, in addition, confers on the insect the capacity to absorb diluted nutrients in the diet [2,13]. In many hymenopteran larval forms, the midgut forms a blind sac. This adaptation enhances nutrient absorption by preventing mixing with faecal matter [14]. Scientists agree that this trait in Hymenoptera has evolved in many ways across different lifestyles. No matter where the larva lives, inside plants (Cynipidae, Tenthredinidae and Eulophidae), inside a host as parasitoid wasps (Braconidae or Ichneumonidae), or in a colony as social insects (Apidae and Vespidae), keeping metabolic waste separate is essential for survival [4,15]. In contrast, in adults, the gastrointestinal tract is continuous, facilitating the processing and excretion of food waste and supporting a more active lifestyle [16,17].

The Cynipidae family comprises phytophagous, gall-inducing wasps. Their larvae feed internally on the nutritive tissues of the plant galls they induce [18,19,20,21,22,23,24,25]. Although these larvae have abundant food resources, their diet is often nutritionally imbalanced compared with the more varied diets of other insect larvae. As a result, Cynipid larvae feed frequently and in large volumes. Gall development is a complex biological process in which the larva manipulates the host plant’s developmental and metabolic pathways [12,19,26,27,28,29,30,31,32,33,34,35,36,37,38,39,40,41]. This manipulation bypasses the plant’s natural defence mechanisms and redirects nutrients from plant growth to support larval development within a specialised microniche. Research on the molecular and biochemical mechanisms of gall wasps, such as *Andricus quercustozae* (Bosc, 1792), supports the hypothesis that gall-inducing insects actively produce, recycle, or manipulate phytohormone levels as a central strategy in insect-plant interactions [21,29,42,43,44,45,46,47,48,49]. In more detail, Cynipidae larvae exhibit robust chemical activity due to their blind midgut. Research shows that insect digestive cells absorb free amino acids from plant tissue via specific transporters [50]. These amino acids travel through the larva’s haemolymph to enlarged salivary glands [51,52], where enzymes convert them into auxins and cytokinins to manipulate plant growth, creating a favourable environment for the larva [18,42]. Proteins in plant tissue are broken down into amino acids, which serve as precursors for new hormone synthesis.

While detailed descriptions of Cynipidae galls exist [41,53,54,55,56,57], the molecular mechanisms of gall induction and development [44,58,59,60,61], and transcriptomic studies [62,63,64,65,66,67] are documented, research on larval anatomy and physiology, particularly of the digestive system, remains limited. While comparative studies of Cynipidae have documented larval alimentary canal structure and foregut/midgut configurations for several tribes (Aylacini, Diplolepidini, Synergini), data for Cynipini remain scarce. Together, these data indicate a trend towards simplified foreguts and midguts in larvae, while the anatomy of the continuous gut that develops in adults has been described in more detail [26,31,33,68]. Although histological and ultrastructural studies of the internal organs of Cynipini larvae remain limited, existing research has primarily focused on larval external morphology. Comparative analyses of features such as body-shape variants, mandibular structure, and mouthpart characteristics offer a substantial basis for identifying morphological characters [26].

To address these aforementioned gaps, our study examines the anatomy of the larvae of *A. quercustozae*, a gall wasp in the tribe Cynipini. The aim is to provide new anatomical insights specific to this species, rather than a comprehensive account of all Cynipini. Recent investigations into *A. quercustozae* have explored the structural complexity of galls and the dynamics of the gall wasp–plant interaction with oak *Quercus virgiliana* (Ten.); syn. *Q. brachyphylloides* Vuk. [41]. These galls are organised into specialised concentric layers. The inner gall contains a nutrient-rich cambial zone and nutritive tissue for the larva, while the outer gall comprises parenchymatous tissue and a protective epidermis. Gall initiation follows a three-stage progression: differentiation and growth, maturation, and lignification, as described by Puljas et al. [41]. Oviposition initiates gall formation, and larval feeding maintains it by stimulating the continuous production of nutritive cells, progressive hyperplasia and hypertrophy of parenchymal tissues, and the addition of new vascular bundles that establish a dedicated nutrient supply to the developing larva. Ultimately, the plant is induced to construct a fortified, vascularised chamber that provides a constant food supply and, subsequently, a lignified shell for safe overwintering during diapause.

Generally, gall formation relies on intricate interactions among insect-derived chemical signals, plant hormones, and specific proteins. Desnitskiy et al. [24] confirmed that insect-derived phytohormones both initiate and sustain gall growth, with gradients in hormone levels between larval tissue and the gall driving this process [49]. These findings support the necessity for a continuous supply of phytohormones during gall development [21,58]. Research has found elevated levels of these phytohormones in the saliva, bodies, or glands of many gall-inducing insects [12,21,39,43,47,69], supporting the idea that the larvae are the source of these hormones in galls. Authors report that a localised increase in auxins and cytokinins in the gall larval chamber drives hyperplasia and hypertrophy, processes also observed in galls of *A. quercustozae* during growth progression [41]. This process compels the plant to alter its typical developmental programming and form a specialised nutritive microniche. At the molecular level, auxins and cytokinins regulate host cell division and elongation by modulating the expression of genes involved in meristem formation and vascular differentiation [70]. To counter plant defences, it is hypothesised that gall-inducing insects secrete small proteins and enzymes that interfere with jasmonic acid synthesis or signalling, thereby lowering activation of defence-related genes [12]. When defence compounds are diminished, and nutrient flow within the gall is altered, nutrient availability can increase, and tannin toxicity may decrease [40]. The key aspect of this process is how insects acquire phytohormones. The main theories consider three mechanisms: (a) ingestion and sequestration from the host plant, (b) microbial symbiosis, and (c) biosynthesis by insects. Current evidence suggests that insects primarily synthesise these hormones themselves, rather than relying on ingestion/sequestration or microbial assistance [71].

Larval digestion likely plays a key role in gall formation and may provide insight into insect-mediated manipulation of phytohormone dynamics. Comprehensive anatomical and ultrastructural analyses, using light and transmission electron microscopy, are expected to contribute to future biochemical and molecular testing of current hypotheses on the mechanisms of phytohormone acquisition. Therefore, the primary aim of this study is to describe in detail the structure and ultrastructure of the digestive tract in gall wasp larvae.

## 2. Materials and Methods

### 2.1. Larval Collection

Ten growing galls with diameters between 2.0 and 3.0 cm containing larvae were sampled from the asexual generation of the gall wasp *Andricus quercustozae* on oak trees (*Quercus virgiliana*) in the Lepenica region of Central Dalmatia (43°37′31.5″ N 16°06′01.6″ E) between August and October 2025. Collections were conducted at a single site, sampling from one tree approximately five metres tall, from which galls had been observed during an initial field survey. The collection took place under favourable weather conditions, with no precipitation. Galls were removed by hand or with manual or telescopic secateurs. Upon arrival at the laboratory, the diameter of each gall was measured, and the galls were carefully dissected with a razor blade and examined under a Leica TL 5000 stereomicroscope, with documentation provided by a Leica DMC 5400 camera (Leica, Wetzlar, Germany).

Larval length was measured. Two larvae were dissected at room temperature in a droplet of 2.5% glutaraldehyde to isolate the midgut and salivary glands, thereby ensuring immediate primary fixation for transmission electron microscopy (JEM-1400 Flash; JEOL, Tokyo, Japan) and minimizing potential artifacts during subsequent ultrastructural analysis. Eight larvae were injected with formalin-acetic acid-alcohol (FAA) for 24 h for histological analysis. This approach rapidly stabilized internal tissues to prevent cellular degradation and maintained the integrity of the larval body cavity. The FAA solution was prepared with 90 parts 70% alcohol, 5 parts 37% formalin, and 5 parts glacial acetic acid, following the method of O’Brien and McCully [72]. Injections were performed using a 1 cc syringe with a 27G1/2 needle.

### 2.2. Histological Preparations

The injected larvae were dehydrated through a graded ethanol series, cleared with xylene, and embedded in Paraplast Plus^®^ (Surgipath^®^, Leica Biosystems, Richmond, IL, USA). They were serially sectioned with a microtome (Leica, Wetzlar, Germany) at 5–7 μm. After removing the Paraplast Plus^®^ with xylene, the sections were placed on microscope slides and double-stained with haematoxylin and eosin. Sections were washed through a graded ethanol series and cleared with xylene. The dried sections were mounted with Biomount (BioGnost Ltd., Zagreb, Croatia). The sections were analysed using a Digicyte Digital Technologies, Ltd. NE930 microscope (Zagreb, Croatia) equipped with a camera for photo documentation. Length ratios of alimentary canal regions were quantified from mid-longitudinal histological sections of six larvae. Measurements were obtained along the central longitudinal axis of the canal using Motic Images Plus 3.0 (×64) software. The linear dimensions of the foregut, blind midgut, and anal region were measured for each specimen. The obtained mean values were expressed as percentages relative to the total alimentary canal length.

### 2.3. TEM Sections

The larval midgut and salivary gland samples were fixed in 2.5% glutaraldehyde in 0.1 M phosphate buffer, pH 7.4, for transmission electron microscopy. After fixation, the sections were washed in 0.1 M phosphate buffer 2 × 10 min on a shaker, followed by postfixation in 1% osmium tetroxide (OsO4) for 1.5 h. Afterward, the tissue was washed in distilled water 2× for 10 min, and en bloc staining with uranyl acetate was performed overnight at +4 °C. The next day, the samples were first placed in uranyl acetate for 30 min at +4 °C, then dehydrated in ethanol of increasing concentrations (50% to 100%) on a shaker, and finally dehydrated in super-dry absolute ethanol for 20 min on a shaker. Before infiltration with Spurr resin (Sigma-Aldrich Inc., St. Louis, MO, USA), a transition step in propylene oxide for 2 × 15 min on a shaker was performed. The samples were infiltrated with epoxy resin in three steps: 50:50 propylene oxide: resin for 1 h on a shaker; 25:75 propylene oxide: resin for 1 h on a shaker; and finally, pure resin for 1 h. This was followed by embedding the samples in pure Spurr resin in silicone moulds, and subsequently polymerising the blocks in an oven at 65 °C for 48 h.

Semi-thin sections were cut at 500 nm with an ultramicrotome (PowerTome XL, RMC Boeckeler, Boeckeler Instruments, Inc., Tucson, AZ, USA), and the area of interest was chosen for ultra-thin sectioning. Ultra-thin sections were cut at 60 nm using Diatome diamond knives (Diatome Ltd., Nidau, Canton Bern, Switzerland) and placed onto copper grids. The sections were then stained with uranyl acetate and lead citrate for contrast enhancement and examined under a transmission electron microscope (JEM-1400 Flash; JEOL, Tokyo, Japan).

## 3. Results

### 3.1. Gross Anatomy of Larva

**Figure 1:**
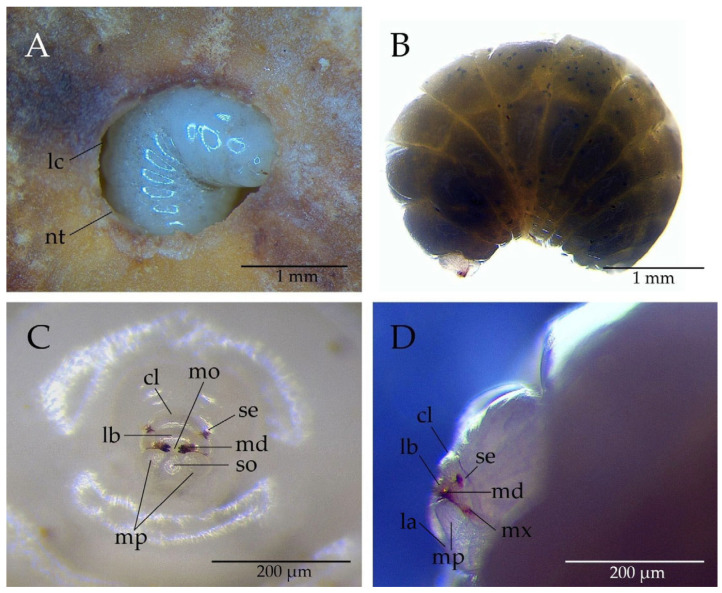
*Andricus quercustozae* larva: (**A**) in a nutrient-dense larval chamber: lc (larval chamber), nt (nutritive tissue); (**B**) whole body; (**C**) head with mouthparts (frontal view): se (setae), md (mandible), so (salivary opening), mp (maxillary palp), lb (labrum), cl (clypeus), mo (mouth opening); (**D**) head with mouthparts (lateral view): se (setae), md (mandible), mx (maxillae), mp (maxillary palp), la (labium), lb (labrum), cl (clypeus).

The diameters of the ten analysed galls ranged from 2.2 to 3.0 cm, with an average of 2.4 cm. Larval lengths were between 2.8 and 4.0 mm, with an average of 3.8 mm. Hymenopteriform in shape, the larva displays clear segmentation and lacks appendages (Figure 1A). Its fusiform, ventrally curved body is white and smooth. Composed of a head and 12 segments, the larva’s overall form is consistent (Figure 1B).

Viewed ventrally, the segments progressively narrow, ending with a shortened last segment at the apex. The head, heart-shaped and protruding ventrally at the mouth region, is distinctive (Figure 1C,D). The clypeus, well-defined and oval, contains a pair of supraclypeal setae. Rectangular and with a concave dorsal surface, the labrum contrasts with the fully exposed mandibles, which have two equally sized teeth. The maxillae rest on the maxillary palps, positioned laterally from the salivary opening, while the rounded labium encircles the salivary opening just above the mouth (Figure 1C,D).

### 3.2. Structure and Ultrastructure of the Larval Alimentary Canal

**Figure 2:**
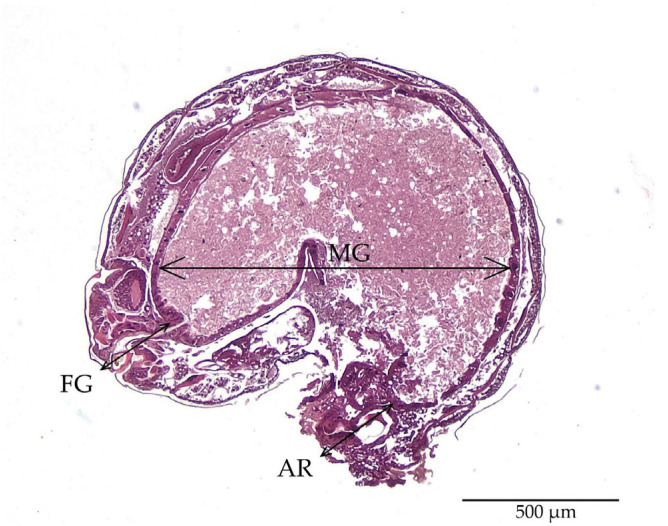
Histological longitudinal section of an *Andricus quercustozae* larva showing regional anatomical specialization and the longitudinal ratio of the alimentary canal: FG (foregut) and AR (anal region) each constitute approximately 10% of the total length, while the blind midgut (MG) accounts for the remaining 80%.

In *A. quercustozoae*, the alimentary canal begins at the mouth and is divided into three morphologically distinct regions: the foregut, a large blind midgut, and the anal region (Figure 2). The foregut constitutes approximately 10% of the total canal length. The midgut, which follows the larval body’s characteristic fusiform profile and displays a pronounced ventral curvature, comprises about 80% of the canal length. The final 10% of the longitudinal axis corresponds to the anal region, which is primarily occupied by the hindgut tube.

#### 3.2.1. Foregut

**Figure 3:**
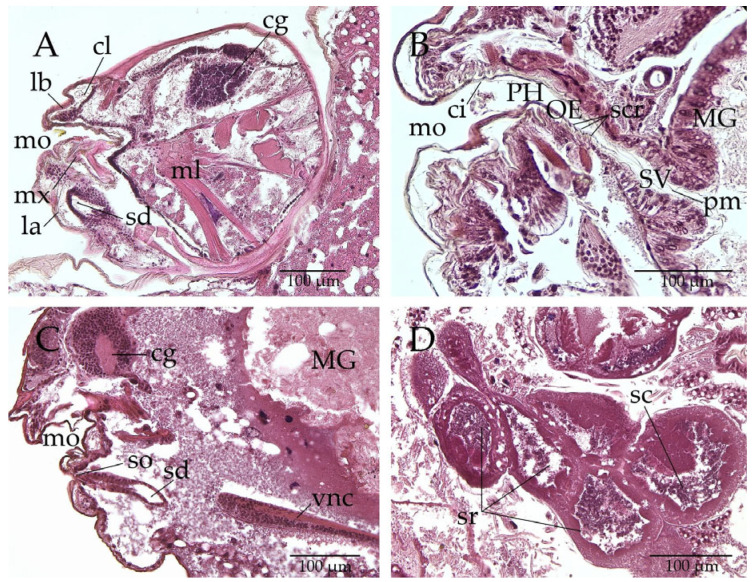
Longitudinal histological section of *Andricus quercustozae* (**A**) head: mo (mouth opening), mx (maxillae), la (labium), lb (labrum), cl (clypeus), sd (salivary duct), ml (muscle), cg (cerebral ganglion); (**B**) foregut: mo (mouth opening), ci (cuticular intima), PH (pharynx), OE (esophagus), scr (sclerotised ridges), SV (stomodeal valve), pm (peritrophic membrane), MG (midgut); (**C**) anterior body: mo (mouth opening), so (salivary opening), sd (salivary duct), cg (cerebral ganglion), vnc (ventral nerv cord); (**D**) salivary gland: sr (secretory regions of the lateral lobe), sc (secretory cavity).

The foregut begins at the mouth opening above the labium, where the salivary gland opening is located (Figure 3A). It is structured as a tube composed of a single-layered epithelium and a cuticular intima. Its primary role is transport through two main structures: the pharynx and the oesophagus (Figure 3B). The pharynx, a short muscular organ, facilitates the passage of food. The oesophagus is a narrow, simple tube that directs food towards the midgut. Despite lacking absorptive cells, the foregut features specialised structures, such as sclerotised ridges on the intima (Figure 3B). The transition between the foregut and midgut is managed by the stomodeal valve (SV), also known as the cardiac or oesophageal valve. This sphincteric structure regulates food flow and contains cylindrical epithelial cells that secrete the peritrophic membrane (Figure 3B)—a semipermeable barrier that protects the midgut epithelium from the abrasive action of food material passing from the foregut.

Paired salivary glands are located ventrally and laterally to the alimentary canal and open in front of the mouth through a common salivary opening (Figure 3A,C). Each gland possesses two long lateral lobes connected to the opening by a pair of salivary ducts (Figure 3C). Each lobe contains multiple secretory regions that release their contents into extracellular secretory cavities (Figure 3D).

#### 3.2.2. Midgut

**Figure 4:**
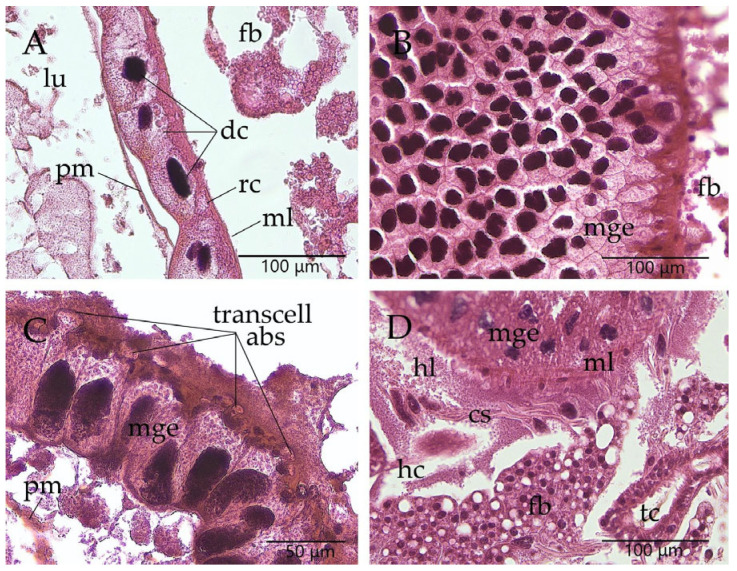
Histological section of *Andricus quercustozae* (**A**) midgut (longitudinal section): dc (digestive cells), rc (regenerative cell), ml (muscle layers), pm (peritrophic membrane), lu (lumen), fb (fat body); (**B**) midgut (transversal section): mge (midgut epithelium); (**C**) midgut (transcellular absorption): mge (midgut epithelium), pm (peritrophic membrane); (**D**) open circulatory system: hc (hemocoel), hl (hemolymph), cs (circulatory system), mge (midgut epithelium), ml (muscle layers), fb (fat body), tc (trachea).

The midgut is a large blind tube composed of a single-layered epithelium, muscle layers (inner circular and outer longitudinal), and a peritrophic membrane that sur-rounds the food bolus within the lumen (Figure 4).

The midgut epithelium is maintained by two specialised cell types: digestive cells and regenerative cells (Figure 4A). Most of the midgut epithelium consists of densely packed, columnar digestive cells with prominent nuclei, commonly called “enterocytes” (Figure 4B). At the cellular level, it seems that absorption is transcellular via transcytosis, meaning the compound is transported across the apical membrane of digestive cells, passes through the cell, and is exported at the basal membrane (Figure 4C). The midgut is surrounded by a hemocoel, an open circulatory system with haemolymph, that helps nourish the organs and fat bodies (Figure 4D).

**Figure 5:**
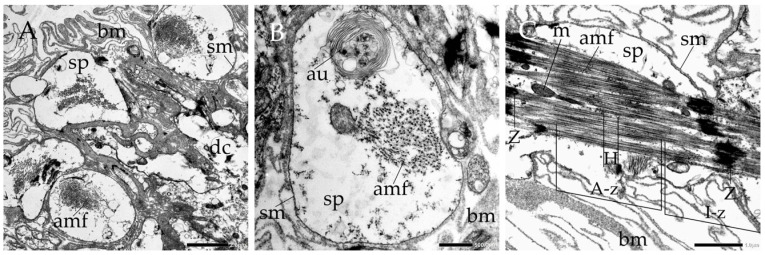
Transmission electron micrographs (TEM) of: (**A**) muscular layer; (**B**) longitudinal muscle; (**C**) circular muscle: bm (basal membrane), amf (actin and myosin filaments) sm (sarcolemma) sp (sarcoplasm), dc (digestive cell), au (autolysosome), Z (Z-lines), A-z (A-zone), I-z (I-zone), H (H-line), m (mitochondria).

**Figure 6:**
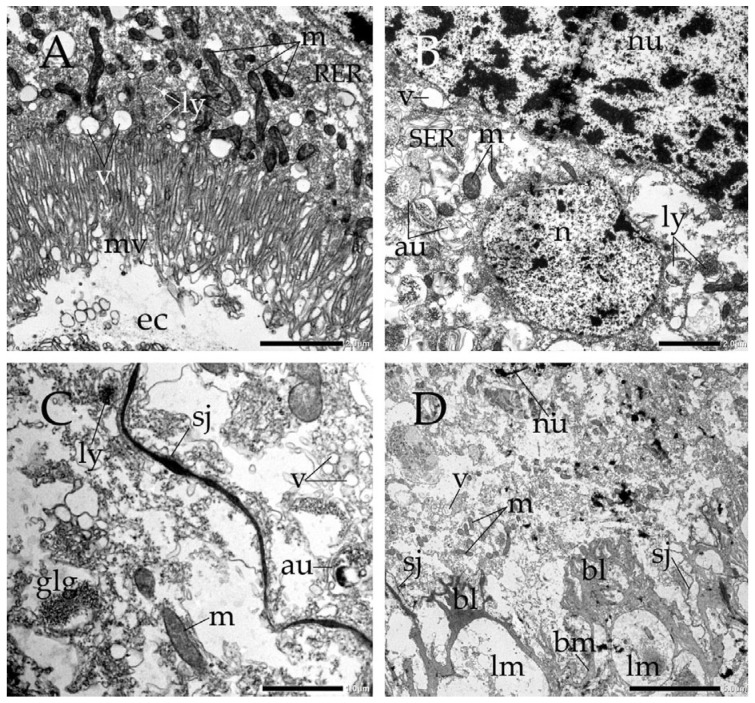
Transmission electron micrographs (TEM) of digestive cell: (**A**) apical region: ec (ectoperitrophic space), mv (microvilli), m (mitochondria), RER (rough endoplasmic reticulum), v (vesicles); (**B**) perinuclear region: nu (nucleus), n (nucleolus), SER (smooth endoplasmic reticulum), au (autolysosomes), m (mitochondria), ly (lysosomes); v (vesicles); (**C**) septate junctions between neighboring digestive cells: sj (smooth septate junction), glg (glycogen granules), ly (lysosome); au (autolysosomes), m (mitochondria), v (vesicles); (**D**) basal region: bl (basal labyrinth), bm (basal membrane), lm (longitudinal muscle), sj (smooth septate junction), nu (nucleus), m (mitochondria), v (vesicles).

Ultrastructurally, the midgut digestive cells exhibit a highly polarised organisation, which appears well suited to intensive nutrient uptake, cellular retention, and transcellular transport within a functionally closed digestive system (Figure 5 and Figure 6). External to the midgut epithelium, an outer longitudinal and an inner circular muscular layer were present (Figure 5). The muscles display striated features, with myofilaments arranged in a regular pattern that likely enables the gut to contract and undergo peristalsis. The circular bands of muscle lie close to the basal membrane (Figure 5A). Actin and myosin filaments are present in both muscle types and are enclosed by the sarcolemma. The part of the cell not occupied by the contractile mechanism constitutes a significant portion of the total volume of the sarcoplasm, in which organelles such as autolysosomes and mitochondria can be observed (Figure 5B,C). The Z-lines that anchor the actin filaments are prominent in circular muscle bands, helping delineate the sarcomere region (Figure 5C). In longitudinal sections of circular muscle, the isotropic zones (areas of actin and Z-lines in the middle) and anisotropic zones (areas of overlap between actin and myosin) can be easily distinguished, as can the H-line, which contains only myosin filaments.

The principal digestive cells of the midgut epithelium demonstrate a distinct structural compartmentalisation, with the apical “brush border” facing the closed gut lumen and the basal domain interacting with the hemolymph cavity. A dense “brush border” of elongated, finger-like microvilli significantly increases the surface area (Figure 6A). A dense population of elongated mitochondria is closely packed immediately beneath this brush border, presumably supplying the ATP required for active, carrier-mediated transport. The ectoperitrophic space, a narrow, fluid-filled region, lies beneath the apical microvilli of the cells (Figure 6A). In the perinuclear region, oval nuclei, typically containing one or two nucleoli, show extensive light euchromatin zones interspersed with massive, peripheral blocks of deeply electron-dense heterochromatin aligned along the nuclear envelope (Figure 6B,C). The surrounding cytoplasmic matrix is granular, rich in free ribosomes, polysomes, and profiles of the rough and smooth endoplasmic reticulum, suggesting high rates of protein and enzyme synthesis. Additionally, it features numerous structures resembling lysosomes, autophagosomes, autolysosomes, multivesicular bodies, glycogen granules, and electron-lucent vesicles, reflecting the closed nature of the digestive tract and a capacity for nutrient sequestration. Smooth septate junctions are present between neighbouring digestive cells (Figure 6C). They act as a tight, cell-sealing barrier that closes the intercellular space, which likely serves as a key pressure-resistant seal that anchors neighbouring enterocytes against the high internal pressure resulting from the absence of an anal opening.

In the basal region, the basal cell membrane forms a network of branching invaginations (basal labyrinth) that extend into the lower cytoplasm (Figure 6D). The close alignment of mitochondria along these membrane folds likely reflects a system designed to support transcellular transport. The surrounding network of transport vesicles and cisternae in the cytoplasm appears to receive, process, and organise newly synthesised compounds, suggesting a pathway that guides these materials toward the hemolymph via transcytosis.

**Figure 7:**
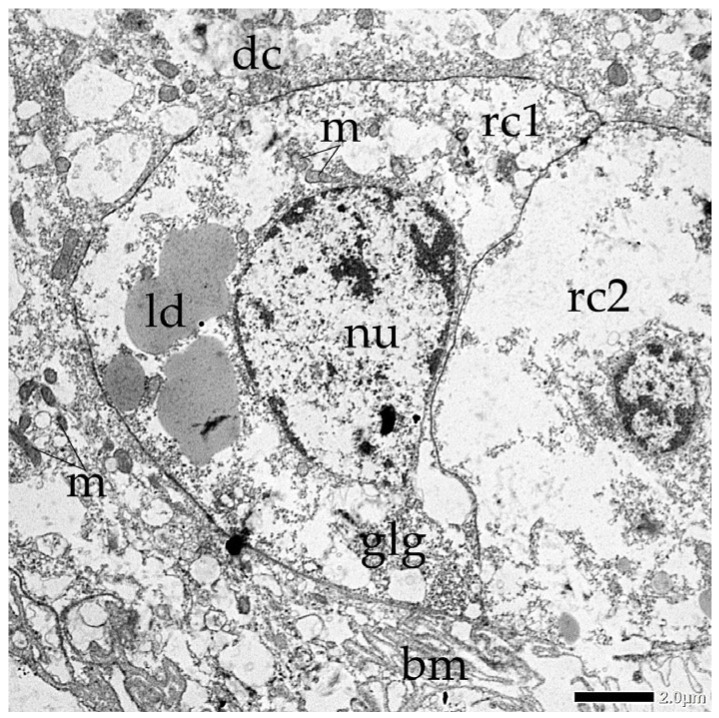
Transmission electron micrographs (TEM) of two daughter regenerative cells (rc1 and rc2) after symmetrical division: nu (nucleus), glg (glycogen granules), ld (lipid droplet), m (mitochondria), dc (digestive cells), bm (basal membrane).

In the basal region of the midgut epithelium, two daughter regenerative cells are closely nested, situated beneath a principal digestive cell. These regenerative cells appear less electronically dense than the surrounding digestive cytoplasm, displaying a relatively clear or translucent matrix. Within the central regenerative cell (rc1), a large, active-looking nucleus occupies most of the cell volume, with prominent patches of condensed heterochromatin. The cytoplasm surrounding this nucleus possesses an assembly of organelles, including small, scattered mitochondria, big lipid droplets and regions filled with glycogen granules. A second regenerative cell (rc2) exhibits similar cytoplasmic clarity and a well-defined nucleus, suggesting a coordinated cluster or nest of undifferentiated cells. The positioning of these cells near the basal labyrinth and basal membrane indicates their role in epithelial renewal, serving as a stem cell niche ready to differentiate and replace senescent enterocytes within the larval midgut (Figure 7).

#### 3.2.3. Anal Region

**Figure 8:**
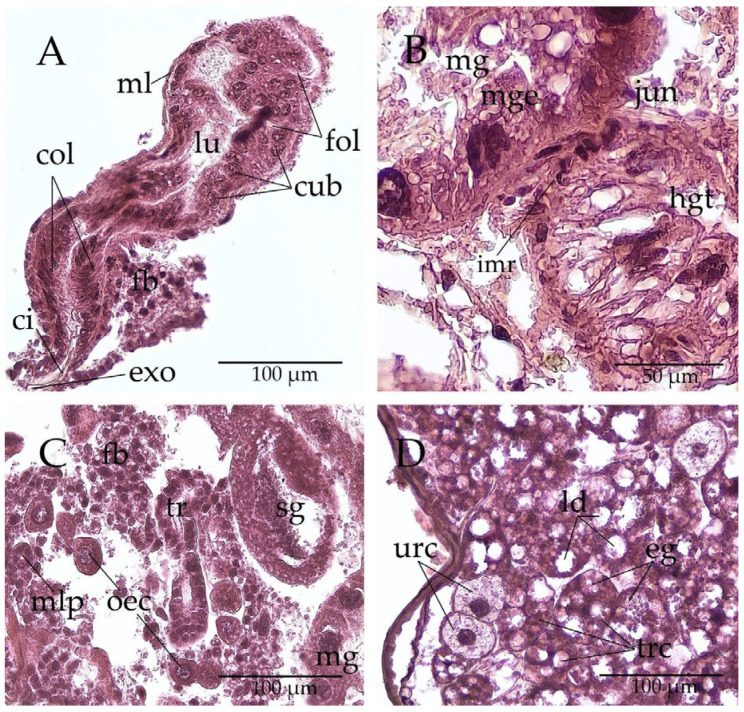
Histological section of *Andricus quercustozae* anal region: (**A**) hindgut tube: cub (cuboidal epithelial cells), col (columnar epithelial cells), ci (cuticular intima), exo (external opening), lu (lumen), fol (horizontal foldings), ml (muscular layer), fb (fat body); (**B**) midgut-hindgut junction: imr (hindgut imaginal ring), jun (midgut-hindgut junction), mg (blind midgut), mge (midgut epithelial cell), hgt (hindgut tube); (**C**) visceral body cavity: oec (oenocytes), mal (Malpighian tubule (transversal section)), sg (salivary gland), mg (midgut), (fat body), tr (trachea); (**D**) perivisceral fat body: trc (trophocytes), ld (lipid droplets), eg (eosinophil protein granules), urc (urocytes).

With a uniquely structured anal region, *A. quercustozae* larvae feature a specialised hindgut tube distinct from the blind midgut (Figure 8), composed of a single layer of columnar or cuboidal epithelial cells with large, spherical, hypertrophied nuclei that occupy more than half of each cell’s volume. The cuboidal epithelium displays horizontal folds that facilitate luminal expansion (Figure 8A). The hindgut tube is surrounded by visceral muscle, ensuring functional integrity. The thin, protective cuticular intima of ectodermal origin covering the luminal face. Since the larvae do not produce bulky digestive waste, the hindgut has an external opening resembling a small anal pore or slit (Figure 8A), functions as a pressure-relief valve or a conduit for specialised chemical signals.

Because the larval midgut is physically separated from the structures of the larval hindgut until the end of the larval stage, primordia of the adult hindgut, i.e., the ring of progenitor cells, serve as precursors of the functional hindgut and continuous digestive tract of the adult insect (Figure 8B). The hindgut-specific imaginal ring is located at the junction where the larval midgut meets the larval hindgut (Figure 8B). The cells within the ring exhibit characteristics of undifferentiated embryonic cells. They are significantly smaller than the surrounding larval cells, and their nuclei occupy most of the cell volume.

In the visceral body cavity, alongside the hindgut tube, the mesodermal fat body is the most prominent structure. Cavity also contains a few Malpighian tubules and the tracheal system (Figure 8C). Within the perivisceral fat body, which is suspended in the hemocoel and supported by the tracheae, two distinct mesodermal cell types can be identified: trophocytes and urocytes (Figure 8D). Urocytes, have a slightly polygonal shape and contain a central nucleus with a significant amount of heterochromatin, along with cytoplasm that contains small vesicles. Trophocytes, the most abundant cell type in the fat body, are rounded and contain nuclei of varying sizes and shapes. Ectodermal oenocytes, cells associated with the fat body, are also abundant (Figure 8C). They have a large, centrally located, rounded nucleus with abundant heterochromatin. Their cytoplasm appears granular and is strongly stained with both hematoxylin and eosin (Figure 8C).

**Figure 9:**
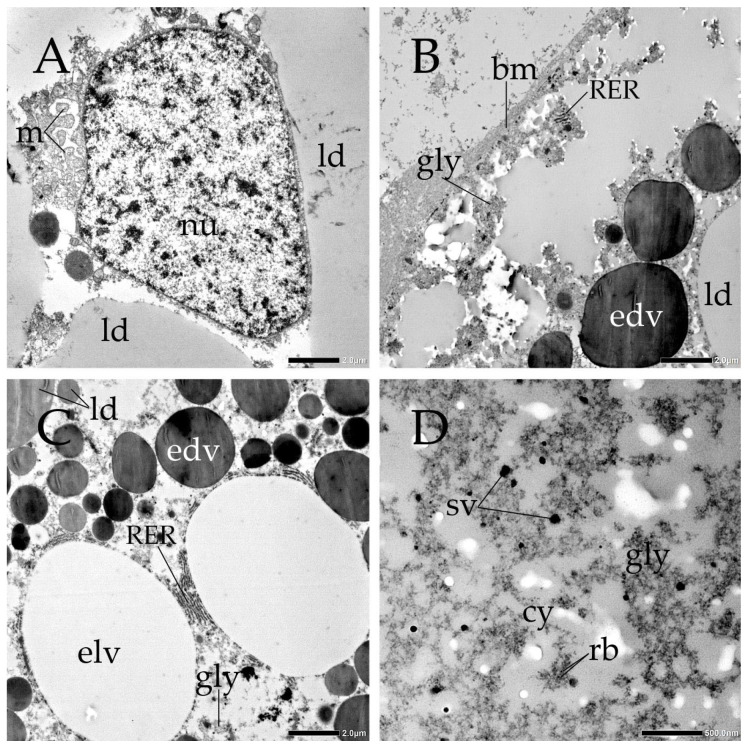
Transmission electron micrographs (TEM) of trophocytes: (**A**) perinuclear region: nu (nucleus), ld (lipid droplets), m (mitochondria); (**B**) peripheral region: gly (islands of glycogen), bm (basal membrane), RER (rough endoplasmic reticulum), ld (lipid droplet), edv (electron-dense vacuole); (**C**) cytoplasm: RER (rough endoplasmic reticulum), elv (electron-lucent vacuole), edv (electron-dense vacuole), gly (islands of glycogen), ld (lipid droplets); (**D**) detailed look at the cytoplasmic substance: gly (islands of glycogen), cy (cytoplasmic matrix), rb (free ribosomes), sv (small electron-dense vesicles).

Ultrastructural analysis of trophocytes revealed that their cytoplasm is packed with large lipid droplets, consistent with energy storage. It also contains electron-dense and electron-lucent vacuoles (Figure 9), which contribute to the pleomorphic or irregular shape of the nucleus (Figure 9A). Dense “islands” of glycogen granules are present and clustered around the cell periphery to facilitate energy mobilisation (Figure 9B). Trophocytes feature a well-developed rough endoplasmic reticulum (RER), indicating active protein synthesis required for the hemolymph (Figure 9C,D).

#### 3.2.4. Salivary Gland

**Figure 10:**
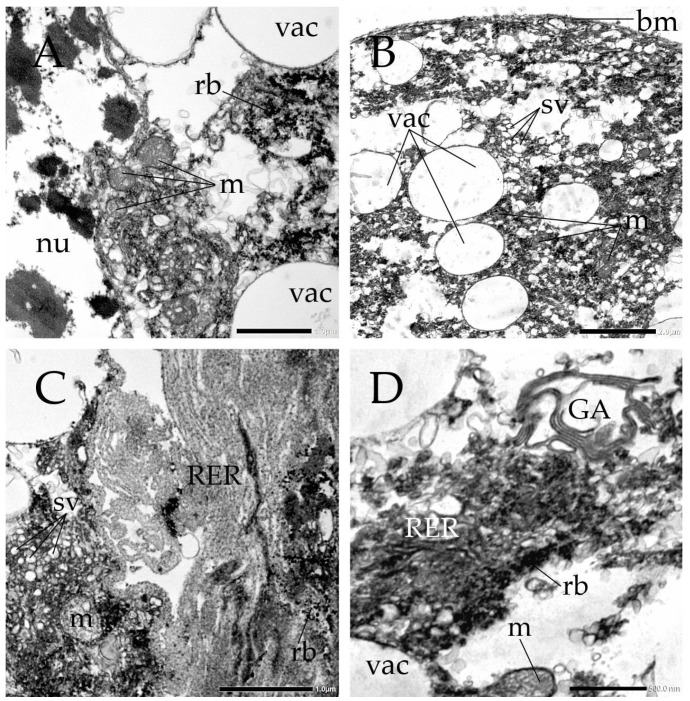
Transmission electron micrographs (TEM) of salivary gland cell: (**A**) perinuclear region: nu (nucleus with condensed chromatin), vac (secretory vacuoles), m (mitochondria), rb (free ribosomes); (**B**–**D**) cytoplasm: vac (secretory vacuoles), sv (small electron-lucent vesicles), m (mitochondria), RER (rough endoplasmic reticulum), GA (Golgi apparatus), rb (free ribosomes), bm (basal membrane).

*Andricus quercustozae* have notably enlarged salivary glands, indicating high secretory activity (Figure 3D). Transmission electron microscopy of a salivary gland cell shows an enlarged, irregular nucleus with condensed heterochromatin (Figure 10A). The cytoplasm contains electron-lucent secretory vacuoles of varying size and internal structure, reflecting different stages of storage or types of secretory products (Figure 10B). Abundant small electron-lucent vesicles and numerous free ribosomes are present. The cells contain a prominent Golgi apparatus and rough endoplasmic reticulum, often organised in dense stacks or concentric arrays (Figure 10C,D). Numerous well-developed mitochondria with parallel tubular cristae provide the ATP needed for active transport.

## 4. Discussion

*Andricus quercustozae* larvae are endophagous herbivores that feed within plant tissue [41]. They develop a specialised alimentary canal adapted to this diet. Our study investigates the internal anatomy of these larvae to elucidate the potential role of their digestive processes in gall formation. Previous anatomical and physiological research has described the general structure of the alimentary canal in Cynipidea. However, the specific morphology and digestive adaptations of *Andricus* larvae remain uncharacterised. As a result, the relationship between their unique digestive features and the mechanisms underlying gall induction and maintenance remains unclear. By providing the first detailed description of the *Andricus* alimentary canal, this study addresses a gap in the literature and clarifies the association between larval digestive processes and gall formation.

Cynipidae larvae primarily feed on the nutritive tissue, an adapted parenchymal layer rich in sugars, amino acids, lipids, and inorganic ions [73]. Research into chemical and genetic mechanisms indicates that insect salivary glands secrete specialised enzymes to extract nutrients from the plant nutritive tissue [74,75,76,77]. In addition, it has been proposed that within the salivary glands, Cynipidae larvae may synthesise phytohormones from free amino acids absorbed through the blind midgut, using them as precursors [21,24,41,49,58,71]. Injection of these hormones into the larval chamber increases the levels of auxin and cytokinin, key regulators of plant growth. Elevated hormone levels are thought to trigger local cell enlargement and rapid division, potentially disrupting normal growth and leading to the development of specialised nutritive tissues. The nutrient-rich fluid from these tissues is then re-ingested. This behaviour is suggestive of a highly specialised adaptation to their ecological niche.

The precise source of phytohormones (such as auxins and cytokinins) in galling larvae remains a topic of debate. While it is possible that, in specific instances, microbial symbionts contribute to gall formation, the evidence argues against microbial symbiosis as a broad source of phytohormones in insects [39]. Consequently, two main possibilities are considered: dietary recycling and endogenous synthesis. This paper explores anatomical features to propose a hypothesis regarding how the cynipid larva’s distinctive digestive and excretory anatomy might function as an integrated biosynthetic system.

The foregut of *A. quercustozae* larvae makes up about 10% of the total alimentary canal length and is relatively simple in structure. The mouthparts—labrum, labium, maxillae, and robust mandibles—are clearly defined and arranged around the mouth opening (Figure 1 and Figure 3). This arrangement suggests an important role in food manipulation [2]. The foregut, supported by sclerotised ridges, forms a single-layered tube that likely transports semi-liquid nutrients from the mouthparts to the midgut (Figure 3B). The muscular pharynx appears to guide food into a narrow oesophagus, which then moves it to the midgut.

The absence of a storage crop in the foregut suggests that the larva can consistently access food within the gall [78]. This anatomical adaptation appears to align with the larval feeding ecology and is accompanied by a substantial expansion of the blind midgut, which occupies most of the visceral cavity and likely serves as the primary site for nutrient storage. The stomodeal valve between the foregut and the midgut is thought to regulate food flow [2]. Epithelial cells at their base secrete the peritrophic membrane, which helps protect the large midgut [1,2].

The blind midgut appears to be specifically adapted for gall life, potentially transforming the digestive tract from a flow-through conduit into a more storage-like reservoir. The large volume of the midgut (Figure 2) provides a morphological indication that larvae might be capable of accumulating high densities of plant nutrients. Based on this observed structure, we hypothesize that the larva may use this space to extract and store precursors for phytohormones, rather than immediately recycling active plant hormones from its diet [48,50]. While further detailed physiological and biochemical studies are strictly required to test this hypothesis, we suggest it is possible that these building blocks are buffered and transported via the hemolymph [44]. The structure of the midgut is consistent with a model in which digestive cells serve as the primary site of enzymatic digestion and nutrient absorption (Figure 4 and Figure 6). The secretory role is characterised by a variable number of granules in the apical cytoplasm, abundant endoplasmic reticulum, and numerous lysosomes, whereas the absorptive role is characterised by long microvilli and elaborate basolateral folds and contains storage products such as glycogen and lipid droplets [4]. The ultrastructure of digestive cells in *A. quercustozoae* larvae supports vesicle-mediated transcytosis as a likely primary transcellular pathway in the midgut epithelium [79]. In Figure 6A, the apical region below the developed brush border accumulates electron-lucent vesicles, which are typical of active endocytotic vesicle formation at the luminal interface. The presence of this vesicle population throughout the epithelium suggests a continuous endomembrane trafficking system [80]. Lysosomes and autophagic structures in the perinuclear cytoplasm (Figure 6B) point to a dynamic vesicle-sorting mechanism. These may process internalised molecules before they reach the hemolymph [81]. The case for transcellular transport is further supported by the presence of extended septate junctions between adjacent digestive cells (Figure 6C). These special junctions seal intercellular spaces, providing a barrier against unregulated diffusion [78]. Molecular complexes or phytohormone-related components are therefore likely to be prevented from passing between cells, necessitating vesicle transport. This energy-dependent process is suggested by the dense packing of mitochondria at the apical periphery (Figure 6A,C) and near cellular boundaries, which supports active vesicle movement [78]. At the basal region of the digestive cells (Figure 6D), the plasma membrane is folded into a basal labyrinth that lies on the basement membrane and adjacent muscles (Figure 5). This expanded basal surface area may indicate exocytotic docking and fusion of intracellular vesicles [4].

We presume that while the blind midgut in hymenopteran larvae (Apocrita) generally functions to maximize nutrient utilization and maintain sanitation [82], this structure has evolved a specialized role in gall-inducing species such as *A. quercustozae*. In these species, the blind midgut likely serves as a high-pressure hormonal reservoir that prevents the rapid loss of phytohormone precursors, thereby optimizing host resource utilization and stabilizing the immediate gall microenvironment. This hypothesis, which requires further detailed physiological and biochemical studies, is additionally supported by the claim that the closed midgut maintains elevated concentrations of phytohormone precursors in the hemolymph, which facilitates their transport to the salivary glands and improves biosynthesis and secretion into the plant [83]. The large midgut epithelium, bounded by a distinct muscular layer (Figure 5), lies within the open haemolymph cavity (Figure 4D), which is filled with circulating haemocytes and provides an immediate pathway across transport barriers [80]. Histological cross-sections suggest an integrated spatial arrangement that appears optimised to facilitate nutrient transfer between the digestive tract and systemic storage tissues (Figure 4 and Figure 8). Generally, insects store dietary nutrients in the haemolymph as proteins, carbohydrates, phospholipids, sugars, or amino acids, and in the fat body as fat, glycogen, proteins, and nitrogenous waste (uric acid) [2]. The vacuolated fat body is in direct contact with haemolymph channels (Figure 4D), minimising the transport distance for mobilised nutrients. To further characterise the presumed storage and synthetic capacity of this enterocyte-fat body axis, the ultrastructure of the metabolic trophocytes was examined using transmission electron microscopy (Figure 9). These mesodermal cells display an active cytological profile centered on a large nucleus and interspersed networks of rough endoplasmic reticulum, consistent with observations of high synthetic capacity in other specialised Hymenoptera [84]. The presence of nearby tracheoles underscores the significant metabolic and respiratory demands at this interface. We observed two additional cell types in the fat body: urocytes and oenocytes (Figure 8C,D). Based on observations, several inferences can be made regarding the role of urocytes and oenocytes in the fat body. First, it can be inferred that urocytes serve as the primary site of “storage excretion,” safely sequestering uric acid as inert crystalline urates. This process could serve as a critical homeostatic mechanism that prevents systemic toxicity and maintains hemolymph pH, indirectly stabilizing circulating phytohormones, which are highly sensitive to internal fluctuations [85]. Furthermore, observed dense clustering of oenocytes in the anal region implies a specialized role in protecting the biochemical integrity of the blind midgut at the site of highest metabolic pressure. It is highly probable that this posterior alignment creates a localized “chemical shield,” generating signals that allow the larva to successfully evade the plant’s immune response [86].

Our findings indicate a specialized hindgut tube morphology (Figure 8) that does not appear to be vestigial. Because it is lined by large cuboidal cells with prominent nuclei, it is possible that this structure possesses secretory or osmoregulatory roles [86], though direct functional evidence is required to confirm this. Additionally, the small excretory opening features a cuticular intima, which could potentially support the biosynthesis theory. While functional testing was not part of this study, we hypothesize that the intima may strengthen the pore, thereby helping it withstand high internal hydrostatic pressure. Under this hypothesis, such an adaptation might enable the opening to function as a pressure-relief valve or a conduit for chemical signals, potentially facilitating the symmetrical spread of signals at the posterior pole. Consequently, we propose that the anatomy of the anal region and hindgut may play a role in maintaining the gall’s geometric precision, representing a compelling avenue for future functional research. In *A. quercustozae*, enlarged salivary glands may indicate increased metabolic activity. Ultrastructural analysis of these glandular cells suggests a potential capacity to synthesize and secrete bioactive compounds, which could support their hypothesized role in influencing host plant tissues during gall formation [49]. The observed ultracellular structures are consistent with the hypothesis that the salivary glands function as a biological sink, potentially allowing the larva to act as a concentrated source of growth regulators (Figure 10). We hypothesize that by sequestering and synthesizing these hormones in specialized glands, the larva might regulate their release to influence host cell division and sustain the gall’s nutritive tissue [18,42]. Furthermore, it is possible that the closed internal system provides the physiological turgor needed to drive the secretion of these hormones through the salivary glands, while the open anal pore and specialized posterior cells may help facilitate the even distribution of signals necessary for symmetrical gall development. However, because direct functional data was not collected in this study, these proposed physiological mechanisms remain tentative hypotheses that require validation through future experimental testing. In conclusion, this paper provides anatomical evidence that allows us to propose a model where the cynipid larva’s unique digestive and excretory anatomy functions as an integrated biosynthetic pump. The morphological evidence presented in this study suggests a potential link between the internal anatomy of *A. quercustozae* and its evolutionary success in transcending the limitations of passive herbivory. We hypothesize that the transport of phytohormones may be managed through a synergy between endodermal and mesodermal tissues. Specifically, the dominance of the midgut, the septate junction-reinforced epithelia, and the extensive basal labyrinth collectively constitute what we interpret as an anatomical hallmark of a high-pressure biosynthetic pump, potentially designed to load endogenously produced signals in mass. The extensive basal labyrinth may serve to amplify the surface area for hemolymph loading, while septate junctions could provide the mechanical integrity necessary to withstand high internal hydrostatic pressure. Within the fat body, it is possible that trophocytes provide energy, urocytes sequester nitrogenous waste, and oenocytes, concentrated in the anal region, refine lipid-phase chemicals to prevent autotoxicity and maintain hemolymph purity. Furthermore, the hindgut tube—which is internally closed but leads to an open external pore impregnated with cuticular intima—is lined with large cuboidal cells that may indicate intense transcriptional activity and the potential production of signaling effectors. Under this proposed framework, the reinforced anal pore could act as a valve regulating internal turgor and facilitating posterior signaling, which may be essential for the geometric round shape of the gall. However, because direct functional testing was outside the scope of this morphological study, these integrated mechanisms are presented as hypotheses to guide future physiological and experimental research.

Cynipid galls are morphologically diverse, showing considerable variation in size, shape, and color, which supports the hypothesis that gall development mechanisms may vary among cynipid wasps [49]. Consequently, conducting future comparative studies of the digestive processes, anatomy, and physiology across different cynipid species that induce diverse gall shapes could significantly enhance our understanding of how digestive system structures might influence gall morphology. Such comparative work will be essential to experimentally validate the functional inferences and anatomical models proposed in this study.

## 5. Conclusions

In conclusion, this study offers an anatomical characterization of the alimentary canal and associated fat body tissues in the *Andricus quercustozae* larva, establishing a structural framework for this gall-inducing species. Our morphological analysis identifies distinct tissue specializations, including an alimentary canal dominated by an expansive blind midgut with a well-developed brush border on the apical surface of the digestive cells, septate junctions, and an extensive basal labyrinth. The enterocyte structure interfaces directly with a vacuolated fat body composed of three cell types: metabolic trophocytes, crystal-bearing urocytes, and a dense posterior cluster of oenocytes near the anal region. The posterior region also contains a non-vestigial hindgut tube lined with large cuboidal cells, terminating at an external anal pore reinforced by a thick cuticular intima. Although these anatomical features—such as reinforced epithelia, expanded cellular surfaces, and localized cell groupings—suggest a capacity for high-pressure containment, metabolic storage, and localized posterior secretion, direct functional data were not collected. These structural findings serve as anatomical baselines and provide a foundation for future physiological, biochemical, and comparative studies across morphologically diverse cynipid galls.

## Data Availability

The raw data supporting the findings of this study will be made available by the first author on request.

## References

[1] Nation J.L. (2008). Insect Physiology and Biochemistry.

[2] Chapman R.F., Simpson S.J., Douglas A.E. (2013). The Insects: Structure and Function.

[3] Chen Y., Li Z., Zhang D., Chen C., Shi J. (2016). Alimentary canal of fifth instar larvae of *Lymantria dispar* (Lepidoptera: Erebidae, Lymatriinae). Entomol. Fenn..

[4] Caccia S., Casartelli M., Tettamanti G. (2019). The amazing complexity of insect midgut cells: Types, peculiarities, and functions. Cell Tissue Res..

[5] Li J., Li C., Wang M., Wang L., Liu X., Gao C., Ren L., Luo Y. (2021). Gut Structure and Microbial Communities in *Sirex noctilio* (Hymenoptera: Siricidae) and Their Predicted Contribution to Larval Nutrition. Front. Microbiol..

[6] Nikelshparg E.I., Bratashov D.N., Nikelshparg M.I., Anikin V.V. Probing Carotenoids in the Gall Wasp *Aulacidea hieracii* in Vivo. Proceedings of the 1st International Electronic Conference on Entomology.

[7] Lucarotti C.J., Whittome-Waygood B.H., Levin D.B. (2011). Histology of the Larval *Neodiprion abietis* (Hymenoptera: Diprionidae) digestive tract. Psyche A J. Entomol..

[8] Bestman J.E., Booker R. (2003). Modulation of foregut synaptic activity controls resorption of molting fluid during larval molts of the moth *Manduca sexta*. J. Exp. Biol..

[9] Kayser H., Palivan C.G. (2006). Stable free radicals in insect cuticles: Electron spin resonance spectroscopy reveals differences between melanization and sclerotization. Arch. Biochem. Biophys..

[10] Rowland I., Goodman W.G. (2016). Magnetic Resonance Imaging of Alimentary Tract Development in *Manduca sexta*. PLoS ONE.

[11] Peng Y.-S., Marston J.M. (1986). Filtering mechanism of the honey bee proventriculus. Physiol. Entomol..

[12] Oliveira D.C., Isaias R.M.S., Fernandes G.W., Ferreira B.G., Carneiro R.G.S., Fuzaro L. (2016). Manipulation of host plant cells and tissues by gall-inducing insects and adaptive strategies used by different feeding guilds. J. Insect Physiol..

[13] Silva C.P., Silva J.R., Vasconcelos F.F., Petretski M.D.A., Damatta R.A., Ribeiro A.F., Terra W.R. (2004). Occurrence of midgut perimicrovillar membranes in paraneopteran insect orders with comments on their function and evolutionary significance. Arthropod Struct. Dev..

[14] Chapman R.F. (1998). Alimentary canal, digestion and absorption. The Insects: Structure and Function.

[15] de Eguileor M., Grimaldi A., Tettamanti G., Valvassori R., Leonardi M.G., Giordana B., Tremblay E., Digilio M.G., Pennacchio F. (2001). Larval anatomy and structure of absorbing epithelia in the aphid parasitoid *Aphidius ervi* Haliday (Hymenoptera, Braconidae). Arthropod Struct. Dev..

[16] Abrunhosa F., Simith D., Monteiro J., Souza A., Oliva P. (2011). Development and functional morphology of the larval foregut of two brachyuran species from northern Brazil. An. Acad. Bras. Ciênc..

[17] Ferrarini M.G., Vallier A., Dell’Aglio E., Balmand S., Vincent-Monégat C., Debbache M., Maire J., Parisot N., Zaidman-Rémy A., Heddi A. (2023). Endosymbiont-containing germarium transcriptional survey in a cereal weevil depicts downregulation of immune effectors at the onset of sexual maturity. Front. Physiol..

[18] Schonrogge K., Harper L.J., Lichtenstein C.P. (2000). The protein content of tissues in cynipid galls (Hymenoptera: Cynipidae): Similarities between cynipid galls and seeds. Plant Cell Environ..

[19] Stone G.N., Schonrogge K., Atkinson R.J., Bellido D., Pujade-Villar J. (2002). The population biology of oak gall wasps (Hymenoptera: Cynipidae). Annu. Rev. Entomol..

[20] Stone G.N., Schönrogge K. (2003). The adaptive significance of insect gall morphology. Trends Ecol. Evol..

[21] Santos J.C., Tavares C.B., Almeida-Cortez J.S. (2011). Plant Vigor Hypothesis refuted: Preference-performance linkage of a gall-inducing weevil on small-sized host plant resources. Braz. J. Biol..

[22] Tooker J.F., Helms A.M. (2014). Phytohormone dynamics associated with gall insects, their potential role in the evolution of the gall-inducing habit. J. Chem. Ecol..

[23] Terra W.R., Ferreira C. (2020). Evolutionary trends of digestion and absorption in the major insect orders. Arthropod Struct. Dev..

[24] Desnitskiy A.G., Chetverikov P.E., Ivanova L.A., Kuzmin I.V., Ozman-Sullivan S.K., Sukhareva S.I. (2023). Molecular aspects of gall formation induced by mites and insects. Life.

[25] Wang Y., Xue C., Wu S., Zhang Y., Li R., Li Y., Yi X. (2025). Transcriptome and phytohormone analysis reveal mechanism of gall formation by *Trichagalma acutissimae* larvae on oak leaves. Front. Plant Sci..

[26] Nieves-Aldrey J.L., Vårdal H., Ronquist F. (2005). Comparative morphology of terminal-instar larvae of Cynipoidea: Phylogenetic implications. Zool. Scr..

[27] Shorthouse J.D., Wool D., Raman A. (2005). Gall-inducing insects—Nature’s most sophisticated herbivores. Basic Appl. Ecol..

[28] Moura M.Z.D., Soares G.L.G., Isaias R.M.S. (2008). Species-specific changes in tissue morphogenesis induced by two arthropod leafgallers in *Lontana camara* L. (Verbenaceae). Aust. J. Bot..

[29] Tanaka Y., Okada K., Asami T., Suzuki Y. (2013). Phytohormones in Japanese mugwort gall induction by a gall-inducing gall midge. Biosci. Biotechnol. Biochem..

[30] Ferreira B.G., Isaias R.M.S. (2014). Floral-like destiny induced by a galling Cecidomyiidae on the axillary buds of *Marcetia taxifolia* (Melastomataceae). Flora.

[31] Polidori C., Nieves-Aldrey J.L. (2014). Diverse Filters to Sense: Great Variability of Antennal Morphology and Sensillar Equipment in Gall-Wasps (Hymenoptera: Cynipidae). PLoS ONE.

[32] Carneiro R.G.S., Isaias R.M.S. (2015). Gradients of metabolite accumulation and redifferentiation of nutritive cells associated with vascular tissues in galls induced by sucking insects. AoB Plants.

[33] Ronquist F., Nieves-Aldrey J.L., Buffington M.L., Liu Z., Liljeblad J., Nylander J.A. (2015). Phylogeny, evolution and classification of gall wasps: The plot thickens. PLoS ONE.

[34] Giron D., Glevarec G. (2014). Cytokinin-induced phenotypes in plant-insect interactions: Learning from the bacterial world. J. Chem. Ecol..

[35] Ferreira B.G., Álvarez R., Bragança G.P., Alvarenga D.R., Pérez-Hidalgo N., Isaias R.M.S. (2019). Feeding and other gall facets: Patterns and determinants in gall structure. Bot. Rev..

[36] Acevedo F.E., Smith P., Peiffer M., Helms A., Felton G.W. (2019). Phytohormones in fall armyworm saliva modulate defense responses in plants. J. Chem. Ecol..

[37] Buffington M.L., Forshage M., Liljeblad J., Tang C.-T., Van Noort S. (2020). World Cynipoidea (Hymenoptera): A key to higher-level groups. Insect Syst. Divers..

[38] Jia M., Li Q., Hua J., Liu J., Zhou W., Qu B., Luo S. (2020). Phytohormones regulate both “fish scale” galls and cones on *Picea koraiensis*. Front. Plant Sci..

[39] Ponce G.E., Fuse M., Chan A., Connor E.F. (2021). The localization of phytohormones within the gall inducing insect *Eurosta solidaginis* (Diptera: Tephritidae). Arthropod-Plant Interact..

[40] Wang W., Guo W., Tang J., Li X. (2022). Phytohormones in galls on eucalypt trees and in the gall-forming wasp *Leptocybe invasa* (Hymenoptera: Eulophidae). Agric. For. Entomol..

[41] Puljas S., Kamenjarin J., Šamanić I. (2026). Structural Complexity of *Quercus virgiliana* Galls Induced by *Andricus quercustozae* (Hymenoptera: Cynipidae). Int. J. Plant Biol..

[42] Mapes C.C., Davies P.J. (2001). Cytokinins in the ball gall of *Solidago altissima* and in the gall forming larvae of *Eurosta solidaginis*. New Phytol..

[43] Straka J., Hayward A., Emery R.J.N. (2010). Gall-inducing *Pachypsylla celtidis* (Psyllidae) infiltrate hackberry trees with high concentrations of phytohormones. J. Plant Interact..

[44] Yamaguchi H., Tanaka H., Hasegawa M., Tokuda M., Asami T., Suzuki Y. (2012). Phytohormones and willow gall induction by a gall-inducing sawfly. New Phytol..

[45] Tokuda M., Jikumaru Y., Matsukura K., Takebayashi Y., Kumashiro S., Matsumura M., Kamiya Y. (2013). Phytohormones Related to Host Plant Manipulation by a Gall-Inducing Leafhopper. PLoS ONE.

[46] Takei M., Yoshida S., Kawai T., Hasegawa M., Suzuki Y. (2015). Adaptive significance of gall formation for a gall-inducing aphids on Japanese elm trees. J. Insect Physiol..

[47] Andreas P., Kisiala A., Emery R.N., De Clerck-Floate R., Tooker J.F., Price P.W., Miller D.G., Chen M.-S., Connor E.F. (2020). Cytokinins Are Abundant and Widespread among Insect Species. Plants.

[48] Tokuda M., Suzuki Y., Fujita S., Matsuda H., Adachi-Fukunaga S., Elsayed A.K. (2022). Terrestrial arthropods broadly possess endogenous phytohormones auxin and cytokinins. Sci. Rep..

[49] Markel K., Novak V., Bowen B.P., Tian Y., Chen Y., Sirirungruang S., Zhou A., Louie K.B., Northen T.R., Eudes A. (2024). Cynipid wasps systematically reprogram host metabolism and restructure cell walls in developing galls. Plant Physiol..

[50] Barroso I.G., Canettieri C.K., Ferreiram C., Terra W.R. (2025). Protein digestion and amino acid absorption mechanisms along the midgut of *Musca domestica* larvae. Comp. Biochem. Physiol. Part B Biochem. Mol. Biol..

[51] Wolfersberger M.G. (2000). Amino acid transport in insects. Annu. Rev. Entomol..

[52] Vårdal H. (2006). Venom gland and reservoir morphology in cynipoid wasps. Arthropod Struct. Dev..

[53] LeBlanc D.A., Lacroix C.R. (2001). Developmental potential of galls induced by *Diplolepis rosaefolii* (Hymenoptera, Cynipidae) on the leaves of *Rosa virginiana* and the influence of *Periclistus* species on the *Diplolepis rosaefolii* galls. Int. J. Plant Sci..

[54] Hernandez-Soto P., Lara-Flores M., Agredano-Moreno L., Jimenez-Garcia L.F., Cuevas-Reyes P., Oyama K. (2015). Developmental morphology of bud galls induced on the vegetative meristems of *Quercus castanea* by *Amphibolips michoacaensis* (Hymenoptera: Cynipidae). Bot. Sci..

[55] Guzicka M., Karolewski P., Giertyczh M.J. (2017). Structural modification of *Quercus petraea* leaf caused by *Cynips quercusfolii*—Histological study of gall. J. Plant Interact..

[56] Ferreira B.G., Freitas M.S., Bragança G.P., Moreira A.S., Carneiro R.G., Isaias R.M. (2019). Enzyme-mediated metabolism in nutritive tissues of galls induced by *Ditylenchus gallaeformans* (Nematoda: Anguinidae). Plant Biol..

[57] Jankiewicz L.S., Guzicka M., Marasek-Ciołakowska A. (2021). Anatomy and ultrastructure of galls induced by *Neuroterus quercusbaccarum* (Hymenoptera: Cynipidae) on oak leaves (*Quercus robur*). Insects.

[58] Bartlett L., Connor E.F. (2014). Exogenous phytohormones and the induction of plant galls by insects. Arthropod-Plant Interact..

[59] Takeda S., Hirano T., Ohshima I., Sato M.H. (2021). Recent progress regarding the molecular aspects of insect gall formation. Int. J. Mol. Sci..

[60] Body M.J.A., Zinkgraf M.S., Whitham T.G., Lin C.H., Schultz J.C. (2019). Heritable phytohormone profiles of poplar genotypes vary in resistance to a galling aphid. Mol. Plant-Microbe Interact..

[61] Roy S., Das A. (2023). Insect-induced foliar galls: A cross-talk among phytohormones for tissue growth and endogenous defense. Khulna Univ. Stud..

[62] Nabity P.D., Haus M.J., Berenbaum M.R., Delucia E.H. (2013). Leaf-galling phylloxera on grapes reprograms host metabolism and morphology. Proc. Natl. Acad. Sci. USA.

[63] Hearn J., Blaxter M., Schönrogge K., Nieves-Aldrey J.-L., Stone G.N. (2019). Genomic dissection of an extended phenotype: Oak galling by a cynipid gall wasp. PLoS Genet..

[64] Schultz J.C., Edger P.P., Body M.J.A., Appel H.M. (2019). A galling insect activates plant reproductive programs during gall development. Sci. Rep..

[65] Takeda S., Yoza M., Amano T., Ohshima I., Hirano T., Sato M.H., Sakamoto T., Kimura S. (2019). Comparative transcriptome analysis of galls from four different host plants suggests the molecular mechanism of gall development. PLoS ONE.

[66] Karinho-Betancourt E., Hernandez-Soto P., Calderon-Cortes N., Rendon-Anaya M., Estrella A.H., Oyama K., Nunez-Farfan J., Valverde P.L. (2020). Ecological genomics of plant-insect interactions: The case of wasp-induced galls. Evolutionary Ecology of Plant-Herbivore Interaction.

[67] Martinson E.O., Werren J.H., Egan S.P. (2022). Tissue-specific gene expression shows a cynipid wasp repurposes oak host gene networks to create a complex and novel parasite-specific organ. Mol. Ecol..

[68] Csóka G., Stone G., Melika G., Raman A., Schaefer C.W., Withers T.M. (2005). Biology, ecology, and evolution of gall-inducing Cynipidae. Biology, Ecology and Evolution of Gall-Inducing Arthropods.

[69] Oates C.N., Denby K.J., Myburg A.A., Slippers B., Naidoo S. (2016). Insect gallers and their plant hosts: From omics data to systems biology. Int. J. Mol. Sci..

[70] Fukuda H. (2004). Signals that control plant vascular cell differentiation. Nat. Rev. Mol. Cell Biol..

[71] Connor E.F. (2026). Insect Production and Secretion of Phytohormones and Impacts on Host Plants. Annu Rev. Entomol..

[72] O’Brien T.P., McCully M.E. (1981). The Study of Plant Structure: Principles and Selected Methods.

[73] Harper L.J., Schönrogge K., Lim K.Y., Francis P., Lichtenstein C.P. (2004). Cynipid galls: Insect-induced modifications of plant development create novel plant organs. Plant Cell Environ..

[74] Ma R., Reese J.C., Black W.C.I., Bramel-Cox P. (1990). Detection of pectinesterase and polygalacturonase from salivary secretions of living green bugs, *Schizaphis graminum* (Homoptera: Aphididae). J. Insect Physiol..

[75] Cherqui A., Tjallingii W.F. (2000). Salivary proteins of aphids, a pilot study on identification, separation and immunolocalization. J. Insect Physiol..

[76] Chaudhary R., Atamian H.S., Shen Z., Briggs S.P., Kaloshian I. (2015). Potato aphid salivary proteome: Enhanced salivation using resorcinol and identification of aphid phosphoproteins. J. Proteome Res..

[77] Liu X., Zhou H., Zhao J., Hua H., He Y. (2016). Identification of the secreted watery saliva proteins of the rice brown planthopper, *Nilaparvata lugens* (Stål) by transcriptome and shotgun LC-MS/MS approach. J. Insect Physiol..

[78] Lehane M.J., Billingsley P.F. (1996). Biology of the Insect Midgut.

[79] Kemmerer M., Bonning B.C. (2020). Transcytosis of *Junonia coenia* densovirus VP4 across the gut epithelium of *Spodoptera frugiperda* (Lepidoptera: Noctuidae). Insect Sci..

[80] Wu K., Li S., Wang J., Ni Y., Huang W., Liu Q., Ling E. (2020). Peptide Hormones in the Insect Midgut. Front. Physiol..

[81] Kunz D., Oliveira G.B., Uchôa A.F., Samuels R.I., Macedo M.L.R., Silva C.P. (2017). Receptor mediated endocytosis of vicilin in *Callosobruchus maculatus* (Coleoptera: Chrysomelidae) larval midgut epithelial cells. Comp. Biochem. Physiol. Part B Biochem. Mol. Biol..

[82] Wharton R., Vilhelmsen L., Gibson G.A.P. (2004). Characterizing basal apocritans (Hymenoptera: Apocrita). Horae Soc. Entomol. Ross..

[83] Ali D. (1997). The Aminergic and Peptidergic Innervation of Insect Salivary Glands. J. Exp. Biol..

[84] Makki R., Cinnamon E., Gould A.P. (2014). The Development and Functions of Oenocytes. Annu Rev. Entomol..

[85] Roma G.C., Bueno O.C., Camargo-Mathias M.I. (2010). Morpho-physiological analysis of the insect fat body: A review. Micron.

[86] Anstee J., Phillips J. (1987). Mechanisms and Control of Reabsorption in Insect Hindgut. Adv. Insect Physiol..

